# Clinical Metabolomics and Nutrition: The New Frontier in Neonatology and Pediatrics

**DOI:** 10.1155/2014/981219

**Published:** 2014-08-27

**Authors:** Angelica Dessì, Flaminia Cesare Marincola, Alice Masili, Diego Gazzolo, Vassilios Fanos

**Affiliations:** ^1^Neonatal Intensive Care Unit, Puericulture Institute and Neonatal Section, Azienda Ospedaliera Universitaria, University of Cagliari, SS 554, Monserrato, 09042 Cagliari, Italy; ^2^Department of Chemical and Geological Sciences, University of Cagliari, Cittadella Universitaria, SS 554, km 4.5, Monserrato, 09042 Cagliari, Italy; ^3^Department of Maternal, Fetal and Neonatal Health, C. Arrigo Children's Hospital, Spalto Marengo 46, 15100 Alessandria, Italy

## Abstract

In the pediatric clinic, nutritional research is focusing more and more on preventing the development of long-term diseases as well as supporting the repair processes important in the therapy of already fully developed diseases. Most children who are hospitalized or affected by chronic diseases could benefit from specific and careful attention to nutrition. Indeed, the state of nutrition modulates all body functions, including the different metabolic processes which, all together, have a profound effect on the development of the health and future of all individuals. Inappropriate food, even in the first periods of life, can accelerate the development of chronic metabolic diseases, especially in the pediatric age. To gain further insights into metabolic cycles and how they are connected with diet and health, nutrition and metabolomics interact to develop and apply modern technologies for metabolic assessment. In particular, nutritionists are evaluating the metabolomic approach to establish the single nutritional phenotypes, that is, the way in which diet interacts with individuals' metabolisms. This strategy offers the possibility of providing a complete definition of the individual's nutritional and health status, predict the risk of disease, and create metabolomic databases supporting the development of “personalized nutrition,” in which diet is attuned to the nutritional needs of individual patients.

## 1. Introduction

Metabolomics, one of the most recent “omics” sciences, can be defined as an approach based on the systematic study of the complete set of metabolites (metabolome) present in a given biological system, whether fluids, cells, or organisms [1]. The metabolome represents the complete set of low molecular weight (typically < 1500 Da) metabolites produced by an organism, which are the end products of gene expression. Thus, it can be viewed as a mirror that reflects the physiological, evolutionary, and pathological state of a biological system. By measuring the metabolome, metabolomics allows us to photograph the genome in its interaction with the environment and thus investigate the metabolic status of an organism in determined physiological conditions as a consequence of drug treatment, environmental influences, nutrition, lifestyle, genetic effects, and so on. Metabolomic analyses can generally be classified as targeted or untargeted. Targeted analyses focus on the characterization of a specific class of metabolites and are used to measure the concentration of a limited number of known metabolites precisely. This approach is important for assessing the behavior of a specific group of compounds in the sample under given conditions. Untargeted metabolomics focuses on the analysis of the metabolome profile to obtain fingerprints without attempting to identify or precisely quantify all the metabolites in the sample. This approach is most useful in biomarker discovery, diagnostics, and revealing specific metabolic patterns of disease. The study of the complex metabolic fingerprint and the comparative analyses of metabolomes are performed by a combined approach of spectrometric and spectroscopic techniques and computer programs. The techniques commonly employed are nuclear magnetic resonance (NMR) spectroscopy, gas or liquid chromatography-mass spectrometry (GC-MS and LC-MS), Fourier transform infrared spectrometry, and capillary electrophoresis-MS (CE-MS). One of the difficulties with all these techniques is that they provide complex datasets, due to the large numbers of metabolites generated by multiple subjects. Thus, the analysis and interpretation of data relies on pattern recognition and discriminant analysis techniques [2]. One of the areas of greatest interest and in which metabolomics has revealed its great potential is that of nutrition. This is at the origin of nutrimetabolomics or nutritional metabolomics, that is, the study of the human or animal metabolome as a function of nutritional status or as a function of a nutritional challenge. Nutrimetabolomics, together with nutrigenomics, is the foundation on which personalized diets are shaped and planned: through the study of the metabolome it is possible to assess changes induced by diet in gene expression and thus, by modifying the nutriments and biomolecules assumed in the diet, we can intervene in the interaction between nutrients and the human metabolism to reach and maintain the best state of health [3, 4]. Thanks to metabolomics, it appears to be possible to assess an individual's state of nutrition to understand how single nutrients influence metabolic regulation and thus to formulate personalized diets which, if followed at an early age, may prevent the onset of certain chronic diseases such as diabetes, inflammatory diseases, and obesity. Nutrimetabolomics appears to be a promising technique also in pediatric and neonatal research. Studies in this field are evolving continuously towards an improvement in pediatric and neonatological practices in hospitals and clinics. It has been found more and more that exogenous factors such as nutrition, especially in the early stages of life, are of fundamental importance owing to their impact on the proper growth of children and their possible implication for diseases in adulthood, in the case of an inappropriate diet [5]. Comprehension of changes in metabolic profiles during one's lifespan, starting from the earliest stages, may represent an important point of reference in arriving at an understanding of their fundamental mechanisms and their consequent metabolic alterations. This review focuses on the applications of metabolomic technology in the context of neonatology and pediatrics with emphasis on the potential preclinical and clinical applications of this technology in the area of nutrition.

## 2. Metabolomics and Nutrition in Neonatology

In the last ten years, many steps forward have been taken by experts in the field of nutrition in identifying nutrients essential for the growth and good health of the newborn. Milk is a key component in the diet of infants all over the world and especially breast milk is the primary source of nutrition for neonates. Human breast milk (HBM) is a complex biological fluid capable of satisfying the nutritional requirements of a rapidly growing child. Besides the normal nutritional substances such as proteins, carbohydrates, fats, vitamins, and minerals, HBM also contains many other biologically active components such as growth factors, antimicrobic compounds, and immunostimulating components that educate neonates' immune system and protect them against pathogens [6, 7]. Many studies have demonstrated the advantages of breastfeeding, especially in the case of preterm neonates who, because of their low weight at birth, are more exposed to the onset of diseases, both in the first months of life and in adulthood [8, 9]. For these patients, it is thus important to have the best possible nutritional support to ensure proper growth and long-term effects on health and wellbeing. Recently, NMR-based metabolomic studies have been performed to better understand the nutritional properties of HBM. The first investigation was carried out by Marincola et al. [10] who compared the metabolic profiles of the hydrosoluble extract from breast milk of mothers who gave premature birth (between 26 and 36 weeks of gestation) with those of formula milk. A detailed NMR spectral analysis of the human milk oligosaccharides (HMOs) was performed by Praticò et al. [11]. Finally, Smilowitz et al. [12] investigated the interindividual variation in the milk metabolome in terms of maternal secretor status, phenotype, and diet. All of these studies pointed out the contribution of several factors to milk composition variation, among which time of collection, mother's diet and length of pregnancy.

**Figure 1:**
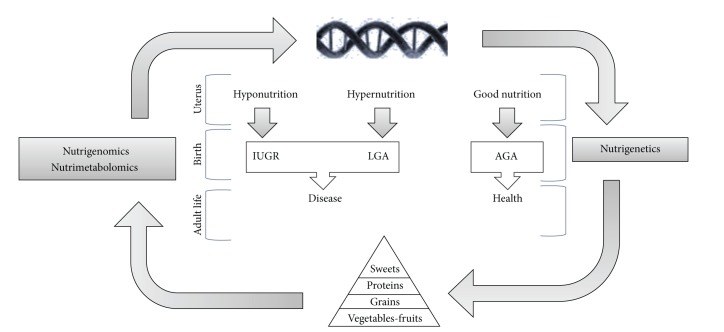
Nutrimetabolomics, nutrigenomics, and nutrigenetics in the life cycle: between genetics and environment and between hypo- and hypernutrition.

At present, nutritionists and dietologists are turning their attention to the discovery of new ways to treat or prevent diseases connected with nutrition such as obesity, diabetes, cardiovascular diseases, and the metabolic syndrome. Several studies have demonstrated that certain chronic pathologies are caused not only by postnatal conditions but also by exposure to epigenetic factors that may influence and permanently alter fetal “programming” [13–15]. It would in fact appear that fetal malnutrition, both excessive and insufficient, may permanently alter the metabolic processes of the fetus and increase the risk of chronic diseases in adulthood [5]. An altered glucid metabolism during fetal development in neonates with intrauterine growth retardation (IUGR) has recently been suggested to be reflected by the increase in extracellular myoinositol that may be considered a valid predictive marker of the development of obesity and type 2 diabetes (T2D) in adulthood [16, 17]. Inositol is in fact known as a secondary messenger of the insulin transducer signal; it is also known that insulin plays a role in favoring lipid and provide synthesis as well as cell growth [18]. In the case of fetal malnutrition (both too much and not enough), it is thus possible to hypothesize that at birth there is a situation of reduced sensitivity to insulin which may be revealed by an increase in extracellular inositol. Recently, the metabolomics approach has been adopted to analyze the urine NMR profiles in large-for-gestational-age (LGA) and IUGR neonates and to define the metabolic patterns associated with such pathologies [19]. The metabolomic analysis made it possible to identify molecules responsible for the different metabolic profiles of IUGR and LGA newborns with respect to a control group. Among these metabolites, myoinositol stood out, whose urine content was higher in both IUGR and LGA neonates. This finding appears to confirm the fact that exposure to a hyperglycemic or hypoglycemic environment in the uterus leads to a common condition of reduced glucid tolerance at birth which tends to persist during growth and into adulthood and consequently to a higher risk of developing pathologies such as obesity and T2D (Figure 1). Thus, comprehension of changes in metabolic profiles during one's lifespan, starting from the earliest stages, may represent an important point of reference in arriving at an understanding of their fundamental mechanisms and their consequent metabolic alterations. Indeed, if it is true that genetics regulates an individual's response to food (nutrigenetics), it is being more and more affirmed that nutrients can control the gene expression (nutrigenomics) and products of the metabolism (nutrimetabolomics).

## 3. Metabolomics and Nutrition in Pediatrics

A number of studies applying metabolomics to nutritional research in pediatrics have been performed (Table 1). Bertram et al. [20] were the first to demonstrate the potential of NMR-based metabolomics in identifying the overall biochemical effects of consumption of different animal proteins in children. In their work the authors investigated the biochemical effects and metabolic differences linked with assumption of large amounts of milk or meat proteins for a limited period of time in children eight years of age. They discovered that the diet based on milk protein generated an increase in urinary excretion of hippurate (mainly derived via gut microfloral breakdown of plant phenolics and aromatic amino acids), thus demonstrating alterations in gut microflora. Differently, the meat diet caused increased urinary excretion of creatine and histidine, whose presence is consistent with the fact that meat is the primary creatine and histidine source, and urea. The NMR analysis of serum revealed a slight influence of the milk diet on the lipid profile, while the meat diet appeared not to have any effect on the serum metabolic profile. These findings confirmed that the metabolomic phenotype is the result not only of genetic factors and diet, but also of the individual's intestinal microbiome. How nutritional habits interfere with the intestinal microbiota is far from understood. The human body contains thousands of millions of microorganisms. They are greater in number than the cells and represent about 3% of body mass. For example, the bacteria that live in the intestinal tract make it possible to digest food and absorb nutritional substances by decomposing most of the proteins, lipids and carbohydrates in our diet, which otherwise would not be assimilated. The microbiome is able to contribute to human survival, with a larger number of genes compared to those of humans themselves. Several experts have hypothesized that a person's intestinal flora has a specific metabolic efficiency and that certain characteristics of microbiome composition may predispose towards a group of obesity-related metabolic abnormalities that increase an individual's risk of developing type 2 diabetes and cardiovascular disease [21]. Even though the microbiome of each individual begins at birth, it is possible to modify its composition through variations in the diet. It has been demonstrated that the kind of diet is capable of modifying certain risk factors for the development of the metabolic syndrome in obese individuals by causing variations in the composition of the bacterial population. In the light of a relationship among obesity, microbiome, and major susceptibility to the metabolic syndrome, it is thus clear that diet assumes a central role in modulating the risk of the disorder.

Infantile obesity is one of the most frequent problems in the pediatric age. The risk of an obese child becoming an obese adult increases with age and is directly related to the amount of excess weight. Moreover, an association between a rapid weight increase in early infancy and an increased risk of obesity in adulthood has been found [22]. Since obesity predicts both short- and long-term adverse health outcomes, including T2D, cardiovascular diseases, hypertension, certain forms of cancer and other obesity-associated problems, research into early-life determinants of obesity could lead to innovative strategies for prevention. Different metabolomic investigations both on animals and humans have been performed on the metabolic state of obese children. As will be shown shortly, since growth might have an impact on metabolism, data in the literature on metabolomic changes related to obesity show some inconsistencies, especially when a comparison between adults and children is made. Mihalik et al. [23] performed a target metabolomic study to determine whether or not obese youth with or without T2D would show the fasting plasma metabolic signatures of elevated amino acid (AA) and medium- to short-chain acylcarnitine (AcylCN) species reported in adults. To this end, tandem mass spectrometry (MS/MS) was used to assess the concentration of AcylCN species and AA in plasma of obese (OB), normal weight (NW), and T2D obese adolescents. The findings demonstrated that long-chain AcylCNs were similar in the three groups. Differently, short- and medium-chain AcylCN and AA concentrations were lower in the plasma of both the OB and the diabetic youths. Furthermore, OB adolescents with T2D demonstrated lower concentrations of the later *β*-oxidation intermediates along with higher rates of fat oxidation. Contrary to what has been reported for adults, the absence of defective fatty acid or amino acid metabolism in OB and T2D adolescents compared with NW support the hypothesis that adolescents and young adults with obesity and T2D have not yet developed the mitochondrial defects that are documented in older adults, and they may even have enhanced mitochondrial activity as an adaptation.

Plasma samples from normal weight, overweight and obese children were profiled by Zeng et al. [24] using GC-MS. Several metabolites (isoleucine, glyceric acid, serine, 2,3,4-trihydroxybutyric acid, and phenylalanine) were screened as potential biomarkers of childhood obesity. Moreover, the waist-hip ratio together with total triglycerides, total cholesterol, high density lipoprotein and low density lipoprotein were suggested to be the most important parameters that correlated with the metabolic disturbances of childhood obesity. Wahl et al. [25] analysed serum samples of OB and NW children between 6 and 15 years of age by using a MS-based metabolomics approach. Metabolite concentrations and metabolite ratios were compared between the two groups of children as well as between children of different pubertal stages. Fourteen metabolites and 69 metabolite ratios were found to be significantly different in OB compared to NW children. In particular, the observed higher concentrations of the acylcarnitines in OB compared to NW were consistent with findings in adults. Differently, a significant reduction of glutamine, methionine, proline, and acyl-alkyl phosphatidylcholine (PC) concentrations was found in OB compared to NW children. Furthermore, OB children exhibited significantly decreased concentrations of the unsaturated lysophosphatidylcholines (LPCs) and increased ratios between saturated LPCs and phatidylcholines. The identified biomarkers were indicative of oxidative stress and changes in sphingomyelin metabolism, in *β*-oxidation, and in pathways associated with energy expenditure.

In the search for weight loss predictors, Wahl et al. [26] analyzed the MS serum metabolite profile of obese children during the Obeldicks lifestyle intervention program, a one-year weight loss program tailored to obese children aged 6–15 years and based on physical activity, nutritional education and behavior therapy that includes individual psychological care of the child and his/her family. Eighty obese children completed the Obeldicks program and were analyzed: 40 achieved a substantial reduction of their body mass index standard deviation score (BMI-SDS) during this program and 40 did not improve their overweight status. The combination of MS data with anthropometric and clinical parameters highlighted long-chain unsaturated phosphatidylcholines and smaller waist circumference as significant predictors of BMI-SDS reduction during the study, thus suggesting a role of phosphatidylcholine metabolism and abdominal obesity in body weight regulation. To enhance the understanding of relationships between obesity and gut microbiota, lifestyle and genetic background in humans, some animal species have been evaluated as experimental models for obesity research. Because of similarities in nutrition and metabolism between pigs and humans [12, 13], genetically obese and lean pigs are useful in childhood obesity research in understanding the mechanisms responsible for development of adiposity. He et al. [27] used NMR-based metabonomics to investigate differences in the serum of genetically obese and lean growing pigs to explore the feasibility of using the obese Ningxiang pig as an animal model for childhood obesity research. The results of this study clearly demonstrated marked differences in serum metabolites, hormones and body composition between obese and lean pigs under the same nutritional and environmental conditions. The altered serum metabonome of obese pigs was mainly ascribed to increased lipogenesis, adipose accumulation, reduced conversion of amino acid nitrogen into urea, and reduced protein synthesis. In addition, changes in gut microbiota-related metabolites, including trimethylamine-N-oxide and choline, were observed in sera of obese pigs compared to lean pigs. Most of these changes are similar to those reported for other animal models as well as children and teenagers. These findings justify the use of the Ningxiang pig as an animal model for childhood obesity research.

Increasing rates of obesity in children have focused attention also on the development of type 1 diabetes (T1D). Indeed, the risk of T1D might rise with increased weight gain as a result of increased insulin resistance and additional stress to beta cells [28]. Zuppi et al. [29] were the first to test the potential of NMR-based metabolomics in highlighting differences between urines from children and adolescents with T1D, but without renal complications, and from healthy individuals matched for sex and age. The levels of alanine, lactate, acetate and citrate were significantly higher in the diabetic patients than in controls. No correlation was found between the duration of T1D and the urinary excretion of the different metabolites. This result was hypothesized to reflect the increased glomerular filtration rate and/or modification of the transport mechanism at the tubular lumen in T1D. Changes in the serum metabolome were assessed prospectively in children who later progressed to T1D by Oresic et al. [30]. For this study, ultraperformance liquid chromatography coupled to mass spectrometry (UPLC-MS) was used. The authors compared the serum metabolome from children who, during followup, progressed to type 1 diabetes-associated autoimmunity and, further, to clinical diabetes or who remained permanently healthy and autoantibody negative. The individuals who developed diabetes had low serum levels of succinic acid and PC at birth, reduced levels of triglycerides and antioxidant ether phospholipids throughout the follow up, and increased levels of proinflammatory lysoPCs several months before seroconversion to autoantibody positivity. The appearance of insulin and glutamic acid decarboxylase autoantibodies was preceded by diminished ketoleucine and elevated glutamic acid. These findings strongly suggested that the metabolic dysregulation precedes overt autoimmunity in T1D.

Balderas et al. [31] used capillary electrophoresis with UV detection to reveal differences in urines of diabetic children as compared to controls and to study the effects of a special additive containing rosemary extract, vitamin E and polyunsaturated fatty acids added to their standard diet through the meat. Analysis of urines obtained after a 12-month treatment evidenced clear differences between treated and nontreated diabetic children. Some of the metabolites associated with T1D were identified, among which guanidinoacetate, creatinine, urea, phenyllactate and p-OH-phenyllactate, phenaceturate, and benzoate. To analyze more in depth the metabolic changes characterizing T1D children, Balderas et al. [32] later also investigated the alterations in plasma and urine of T1D diabetic children who were under insulin treatment with good glycemic control. Plasma samples were analyzed with LC-MS, while urines with CE-MS. Compared to the control group, the main changes in plasma of T1D patients were associated with the lipid metabolism and some markers of the differential activity of the gut microflora. Changes in urine composition were associated with the protein and amino acid metabolism. Furthermore, one early glycation end product (fructosamine) was excreted in higher proportion in the diabetic group. A detailed analysis of the NMR-based metabolomics profiles of diabetic and healthy children's urine was presented by Culeddu et al. [33]. By excluding from the statistical analysis the NMR signals of sugars and citrate (related to the carbohydrate metabolism) and of hippurate (a metabolite of bacterial origins) whose presence overwhelmed all the other compound effects on classification models, the authors evidenced other metabolites (p-cresol sulphate and phenylacetylglycine) as relevant biomarkers. These metabolites were suggested to reflect changes in gut microbiota composition of T1D children with respect to the control group. Additional urine biomarkers associated with T1D were found by Deja et al. [34]. They investigated the relation between the level of glycated hemoglobin (HbA1c) and concentration of metabolites in urines of T1D children and teenagers by using 1H NMR target analysis. Differences among patients with HbA1c levels below (L-T1D) and above (H-T1D) 6.5% showed the importance not only of glucose, ketone bodies and HbA1c, but also of other urine metabolites such as alanine, valine, acetate, pyruvate and citrate, probably related to the endogenous glucose production pathway from proteins.

Recently, the biochemical conditions at the root of inflammatory intestinal disease and their impact on the systemic and gastrointestinal metabolism was studied by Baur et al. [35]. The authors monitored the metabolic events associated with the progressive development of Crohn's disease in a mouse model (from the age of 4 up to 24 weeks). The metabolic profiles of different intestinal compartments were generated by combining NMR spectroscopy and LC-MS. Data revealed shifts in the intestinal lipid metabolism concomitant with the histological onset of inflammation. Moreover, the condition of advanced disease was characterized by a significantly altered metabolism of cholesterol, triglycerides, phospholipids and sphingomyelin in the inflamed tissue (ileum) and the adjacent parts of the intestine (proximal colon). Finally, the effects of nutrition on the metabolic and kinetic equilibrium in children affected by the celiac disease (CD) were assessed in a metabolomic work by Sellitto et al. [36]. The authors combined high-resolution culture-independent methods based on pyrosequencing of barcoded 16SrRNA gene amplicons, quantitative PCR and NMR-based metabolomics to determine the composition and temporal changes of the gut microbiota and to identify potential biomarkers associated with onset of CD in infants with genetic predisposition for CD over the first two years of life. The results showed that infants genetically susceptible to CD who were exposed to gluten at an early age mounted an immune response against gluten and developed CD autoimmunity more frequently than at-risk infants in which gluten exposure was delayed until 12 months of age. Furthermore, the metabolomic analysis of fecal samples revealed potential biomarkers for the prediction of the disease.

## 4. Conclusions

On the basis of published studies, it can be stated that metabolomics is showing itself to be a powerful investigative tool in pediatric and neonatological nutrition research and, in particular, in searching for new biomarkers associated with nutritional health and/or disease status. The final goal is to arrive at an understanding of the biological behavior of a cell system in response to outside stimuli and clear the way to a comprehension of the complex network of interactions between nutrients and molecules (nutrimetabolomics). It is the study of cell metabolites of low molecular weight in response to dietetic treatments that will make it possible to design a kind of nutrition tailored to the genes of each individual, especially in the fields of neonatology and pediatrics.

## Figures and Tables

**Table 1:** A summary of metabolomic studies assessing variations in nutritional health and/or disease status in paediatrics.

Disease	Population studied	Samples	Platform	Biological processes associated with the metabolic disorder	Reference
Obesity	Adolescents: (i) Normal weight (*n* = 39) (ii) Obese (*n* = 64) (iii) T2D (*n* = 17)	Plasma	MS/MS	No defects in fatty acid and amino acid metabolism	Mihalik et al. [23]
	Children: (i) Normal weight (*n* = 16) (ii) Obese (*n* = 32) (iii) Overweight (*n* = 13)	Plasma	GC-MS	Changes in lipid metabolism	Zeng et al. [24]
	Children: (i) Normal weight (*n* = 40) (ii) Obese (*n* = 80)	Serum	MS	Oxidative stress, changes in sphingomyelin metabolism, in *β*-oxidation, and in pathways associated with energy expenditure.	Wahl et al. [25]
	Obese children: (i) With substantial overweight reduction (*n* = 40) (ii) Without substantial overweight reduction (*n* = 40)	Serum	MS	Phosphatidylcholine metabolism	Wahl et al. [26]
	Pigs (4 months of age): (i) Obese type (*n* = 10) (ii) Lean type (*n* = 8)	Serum	NMR	Lipogenesis, lipid oxidation, energy utilization and partition, protein and amino acid metabolism, and fermentation of gastrointestinal microbes	He et al. [27]

Diabetes 1	Children: (i) T1D (*n* = 25) (ii) Control (*n* = 25)	Urine	^ 1^H-NMR	Increased glomerular filtration rateand/or a modification of the transport mechanisms at thetubular level.	Zuppi et al. [29]
	Children: (i) Who progressed to T1D (*n* = 50) (ii) Who remained healthy and autoantibody negative (*n* = 67)	Serum	UPLC-MS	Dysregulation of lipid and amino acid metabolism preceding islet autoimmunity	Ore$\overset{ˇ}{\text{s}}$i$\overset{ˇ}{\text{c}}$ et al. [30]
	Children: (i) T1D (*n* = 34) (ii) Control (*n* = 16)	Urine	CE-UV	—	Balderas et al. [31]
	Children: (i) T1D (*n* = 34) (ii) Control (*n* = 15)	Plasma Urine	LC-MS CE-MS	Dysregulation of lipid metabolism and activity of the gut microflora Protein and amino acid metabolism, glycation	Balderas et al. [32]
	Children: (i) T1D (*n* = 19) (ii) Control (*n* = 90)	Urine	^ 1^H-NMR	Carbohydrate metabolism and gut microbial metabolism	Culeddu et al. [33]
	(i) Diabetic children and teenagers with T1D (*n* = 30) (ii) Control (*n* = 12)	Urine	^ 1^H-NMR	Endogenous glucose production pathway from proteins	Deja et al. [34]

Inflammatory bowel disease	Mice (4–24 weeks of age)	Hydrophilic and lipophilic extracts from intestinal compartments	NMR LC-MS	Modifications of the general cell membrane composition, alteration of energy homeostasis, and the generation of inflammatory lipid mediators	Baur et al. [35]

Celiac disease	Infants genetically susceptible for CD (*n* = 47)	Stool	NMR	GI tract microbiota metabolism	Sellitto et al. [36]
